# Melatonin Enhances Glutathione Peroxidase Activity and Improves Antioxidant Defense in Cryopreserved Ovarian Transplants: A Rat Model Study

**DOI:** 10.3390/antiox15050551

**Published:** 2026-04-26

**Authors:** Karla Krislane Alves Costa Monteiro, Luciana Lamarão Damous, Marcos Eiji Shiroma, José Antonio Orellana Turri, Ricardo dos Santos Simões, Manuel de Jesus Simões, José Cipolla-Neto, Lara Termini, Rinaldo Florencio-Silva, Peter Chedraui, Russel J. Reiter, Edmund Chada Baracat, Jose Maria Soares Junior

**Affiliations:** 1 Laboratório de Ginecologia Estrutural e Molecular (LIM-58), Disciplina de Ginecologia, Departamento de Obstetrícia e Ginecologia, Hospital das Clínicas HC-FMUSP, Faculdade de Medicina, Universidade de São Paulo, São Paulo 05403-010, Brazil; 2 Laboratório de Neurobiologia Pineal, Departamento de Fisiologia, Universidade Federal de São Paulo, São Paulo 04039-032, Brazil; cipolla@icb.usp.br; 3 Laboratório de Inovação em Câncer, Centro de Pesquisa Translacional em Oncologia (CTO), Instituto do Câncer do Estado de São Paulo (ICESP), Faculdade de Medicina, Universidade de São Paulo, São Paulo 01246-000, Brazil; 4 Department of Morphology and Genetics/Discipline of Histology and Structural Biology, Escola Paulista de Medicina—Universidade Federal de São Paulo (EPM-UNIFESP), São Paulo 04023-062, Brazil; silva05@unifesp.br; 5 Escuela de Postgrado en Salud, Universidad Espíritu Santo, Samborondón 092301, Ecuador; pchedraui@uees.edu.ec; 6 Department of Cellular and Structural Biology, University of Texas Health Science Center at San Antonio, San Antonio, TX 78229, USA

**Keywords:** cryopreservation, fertility preservation, GPx, VEGF, ovarian transplantation, oxidative stress, angiogenesis, rats

## Abstract

**Background:** Although ovarian cryopreservation is an essential strategy for fertility preservation, ischemia–reperfusion injury and oxidative stress can significantly compromise graft viability after transplantation. Melatonin is a potent antioxidant capable of modulating redox homeostasis and tissue repair; however, its effects on the ovarian microenvironment after cryopreservation are not fully understood. **Objective:** To investigate whether melatonin supplementation during ovarian cryopreservation enhances GPx1/2-mediated antioxidant defense, preserves follicular integrity, and modulates the angiogenic balance (assessed via VEGF-A expression) after autologous ovarian transplantation in rats. **Methods:** Twenty-four Wistar rats were ovariectomized and divided into control (standard cryopreservation) and melatonin-treated (0.1 μM melatonin) groups. Ovaries were cryopreserved, thawed, and autotransplanted. After 30 days, the grafts were analyzed for GPx1/2 expression (immunohistochemistry), VEGF-A levels (ELISA), biochemical markers, and follicular integrity (histomorphometry) **Results:** The melatonin treatment significantly increased GPx1/2 expression in the corpus luteum (*p* = 0.002), theca interna (*p* = 0.007), and interstitium (*p* = 0.012), and reduced the number of degenerated follicles (*p* = 0.03). Although absolute VEGF-A levels did not differ between groups, melatonin-treated animals showed higher VEGF/FSH ratios (*p* = 0.0007) and VEGF/LH (*p* = 0.0494) ratios. Positive correlations were observed between GPx1/2 expression and VEGF-A expression. **Conclusions:** Melatonin increases antioxidant defenses in cryopreserved ovarian grafts through the upregulation of GPx1/2 and preservation of follicular morphology. Instead of directly increasing VEGF-A levels, melatonin appears to modulate angiogenic signaling, contributing to a more stable microenvironment for ovarian graft survival.

## 1. Introduction

The cryopreservation of ovarian tissue is currently considered one of the main strategies for preserving reproductive potential, particularly in patients exposed to gonadotoxic conditions [1,2,3,4,5,6]. However, ovarian graft success remains limited by the ischemia–reperfusion injury and oxidative stress that occur during freezing, thawing, and subsequent transplantation, significantly compromising graft viability and follicular survival [7,8,9].

During cryopreservation, increased oxidative imbalance—driven by reactive oxygen species (ROS)—can impair cellular homeostasis and compromise tissue integrity [7,8,9,10]. This imbalance particularly affects follicular components, including granulosa and theca cells, as well as the corpus luteum [11,12]. Among the endogenous antioxidant defense systems, glutathione peroxidase (GPx) plays a fundamental role in the detoxification of hydrogen peroxide and lipid peroxides, helping to preserve cellular integrity. A reduction in GPx activity has been associated with increased oxidative damage and follicular degeneration in ovarian tissue [13,14,15].

In this context, the evaluation of endogenous antioxidant enzymes, such as GPx, provides an indirect measure of redox balance and cellular defense capacity in ovarian tissue, reflecting intrinsic mechanisms of antioxidant protection against cryopreservation-induced oxidative damage [8,11,13,15,16]. Thus, increased GPx activity may reflect improved redox balance and a potential reduction in oxidative stress at the tissue level.

Melatonin is widely recognized for its antioxidant properties, particularly due to its ability to modulate the redox balance and enhance cellular defense mechanisms [17,18,19]. Experimental evidence from cryopreserved ovarian tissue and reproductive cell models has demonstrated that melatonin improves follicular survival, increases oocyte quality, and aids early embryonic development under oxidative stress conditions. In ovarian grafts, its use has been associated with improved tissue architecture, increased follicular maturation, reduced apoptosis, and modulation of antioxidant enzyme activity, corroborating its protective role during cryopreservation and transplantation [17,20,21]

In addition to its antioxidant effects, melatonin can also influence angiogenic pathways, representing an additional mechanism of tissue protection, especially under the hypoxic conditions that ovarian grafts are exposed to in the early post-transplant period [22]. Vascular endothelial growth factor A (VEGF-A) is a key regulator of angiogenesis and vascular homeostasis, promoting endothelial cell proliferation, increased vascular permeability, and neovessel formation [23,24]. Therefore, VEGF-A assessment can provide information on whether melatonin acts on angiogenic signaling or by directly increasing VEGF-A expression to induce a more stable microenvironment for graft survival [22].

In this context, the present study evaluated the effects of melatonin supplementation during ovarian cryopreservation on antioxidant defenses and the tissue microenvironment after autologous ovarian transplantation in rats. Specifically, we investigated GPx1/2 expression and follicular integrity as primary outcomes, while VEGF-A expression was evaluated as an indicator of potential angiogenic modulation.

## 2. Materials and Methods

### 2.1. Ethical Approval and Sample Size Calculation

The local Ethics Committee on the Use of Animals (CEUA-FMUSP, protocol No. 024/15, dated 25 March 2015) approved the experimental protocols. Based on information from a previously published work assessing melatonin therapy and follicular expression in vitrified ovarian grafts [20], the Altman nomogram [25] was used to calculate the sample size. For two experimental groups, a total of 24 animals were needed to achieve 80% statistical power and a 5% significance level. GPx1/2 immunoreactivity was the main outcome, while VEGF-A expression was evaluated as a secondary outcome related to angiogenic signaling.

### 2.2. Animals and Experimental Groups

A total of 24 female Wistar rats (*Rattus norvegicus albinus*, 3 months old and weighing about 250 g) were included in the study. The animals were obtained from the University of São Paulo’s institutional animal facility. The experimental unit was a single animal. Before the start of the experiments, the animals were acclimated to the laboratory conditions for at least seven days. The animals in the experiment had ad libitum access to food and water and were kept under a controlled “zeitgeber time” (ZT) 12/12 h light/dark cycle [26], with ZT0 being defined as lights on at 6:00 a.m. All procedures were conducted in accordance with institutional and national guidelines for the care and use of laboratory animals, ensuring ethical and humane treatment. The animals were monitored daily for general health, behavior, and postoperative recovery.

### 2.3. Experimental Design and Group Allocation

Animals were randomly allocated into two groups—a melatonin group (MG) and a control group (CG)—using a random number generator. All procedures were performed under standardized laboratory conditions and the animals were housed under identical environmental conditions to minimize confounders.

Due to intraoperative complications, two animals were lost, resulting in a final sample of 20 animals, resulting in *n* = 10 per group. Despite the loss of animals, statistical significance was maintained for the primary outcome (GPx1/2 expression). The inclusion and exclusion criteria were defined a priori. Only healthy three-month-old female Wistar rats with regular estrous cycles were included. Animals which presented severe intraoperative complications or were lost during surgery were excluded. No data points were excluded from the statistical analysis.

Group allocation was not blinded during the surgical procedures. However, the investigators that performed the histological assessments and statistical analysis were blinded to the group allocation.

In the CG, ovaries were cryopreserved using standard slow-freezing protocols with M2 medium, dimethyl sulfoxide (DMSO), and ethyl alcohol as the carrier, according to an established methodology [27]. In the MG, melatonin (0.1 μM; Sigma-Aldrich, St. Louis, MO, USA), dissolved in ethyl alcohol as vehicle, was added to the culture medium following previously described protocols [28,29].

### 2.4. Estrous Cycle Monitoring

Estrus was standardized for surgical treatments, and only animals with regular cycles of 4 to 5 days were employed. In order to characterize the estrous cycle using the Shorr–Harris approach, vaginal smears were first taken every day between ZTs 2 and 4 [26]. ZT stands for zeitgeber time in a 12 h/12 h light/dark cycle; ZT0 represents the change from dark to light and ZT12 represents the change from light to dark. Vaginal swabs were thus taken two to four hours after the lights were switched on.

### 2.5. Oophorectomy and Autologous Ovarian Transplantation

Animals were anesthetized by intraperitoneal injection of xylazine (15 mg/kg) and ketamine (60 mg/kg) [30]. After laparotomy, the ovaries were identified, and their pedicles were clamped and marked with 4-0 nylon sutures. Bilateral oophorectomy was performed at the uterine horn junction, followed by careful hemostasis. The ovaries were washed with 0.9% saline solution and freed from the surrounding periovarian fat and oviducts [17].

The ovaries from both groups were kept in liquid nitrogen for 24 h after being cryopreserved via gradual freezing [10,17,27]. After this period, the samples were thawed at room temperature (25 °C). Without vascular anastomosis, the ovaries were reimplanted in the retroperitoneum of their respective donors, one on each side of the major vessels (aorta and inferior vena cava), and fixed with a simple suture using non-absorbable thread (4-0 nylon).

After surgery, vaginal smears were obtained daily between ZT2 and ZT4 starting on postoperative day 15 and continuing until euthanasia. Animals were euthanized between postoperative days 30 and 35 during the diestrus phase, which was selected as the standardized experimental endpoint to enable consistent ovarian graft collection and blood sampling for the biochemical and histological analyses. The animals were euthanized by decapitation under deep anesthesia in accordance with national and institutional guidelines for the care and use of laboratory animals, including those established by the National Council for the Control of Animal Experimentation (CONCEA) and the Ethics Committee on the Use of Animals (CEUA) of the Federal University of São Paulo (UNIFESP), as well as the recommendations of the AVMA Guidelines for Animal Euthanasia [31,32]. All procedures were performed by trained and qualified personnel, ensuring compliance with ethical standards and minimizing animal suffering. This method was selected to allow for rapid blood collection, reducing hormonal and biochemical changes related to stress.

### 2.6. Blood Collection and Biochemical Analysis

After 30 to 35 days, animals in the diestrus phase were euthanized as described previously (Section 2.5). Blood was immediately collected into tubes without anticoagulant for hormonal determination. After coagulation at room temperature, the samples were centrifuged at 4 °C (1500× *g* for 10 min), and the serum was separated and stored at −80 °C until analysis. Hormonal and biochemical determinations were performed using commercially available enzyme-linked immunosorbent assay (ELISA) kits (Elabscience, Houston, TX, USA), according to the manufacturer’s instructions. All samples were processed under standardized conditions to minimize pre-analytical variability.

The biochemical analyses were performed using commercially available ELISA kits specifically designed for rat samples (Elabscience, Houston, TX, USA), according to the manufacturer’s instructions. The following hormones were measured: follicle-stimulating hormone (FSH; E-EL-R0391; detection range: 3.13–200 ng/mL; sensitivity: 1.88 ng/mL), luteinizing hormone (LH; E-EL-R0026; detection range: 1.56–100 mIU/mL; sensitivity: 0.94 mIU/mL), estradiol (E-OSEL-R0001; detection range: 3.13–200 pg/mL; sensitivity: 1.17 pg/mL), progesterone (E-EL-0154; detection range: 0.31–20 ng/mL; sensitivity: 0.15 ng/mL), anti-Müllerian hormone (AMH; E-EL-R3022; detection range: 62.5–4000 pg/mL; sensitivity: 37.5 pg/mL), and inhibin B (E-EL-R1027; detection range: 15.63–1000 pg/mL; sensitivity: 9.38 pg/mL).

All samples were analyzed in triplicate and processed on the same day to minimize variability between assays. Cross-reactivity with other steroids was reported to be <0.01%. In addition, LH/FSH and FSH/AMH ratios were calculated for further hormonal assessment.

### 2.7. Determination of VEGF-A Levels

Circulating VEGF-A levels were measured in plasma samples using a specific ELISA kit (E-EL-R2603; Elabscience™, Houston, TX, USA), with a detection range of 31.25–2000 pg/mL and sensitivity of 18.75 pg/mL. Samples were processed in triplicate, according to the manufacturer’s instructions.

After obtaining data from each group of animals, the results were subjected to statistical analysis in order to verify the differences between the melatonin-treated and control groups.

### 2.8. Histological and Immunohistochemical Analyses

Ovarian tissues were fixed in 10% phosphate-buffered formaldehyde (pH 7.2) for 24 h, dehydrated in a graded ethanol series, cleared in xylene, and embedded in paraffin. Serial sections (3 μm) were obtained using a rotary microtome (Leica RM2235, Leica Biosystems, Wetzlar, Germany). The sections were stained with hematoxylin and eosin (H&E) for histomorphometric analysis or processed for immunohistochemistry to evaluate GPx1/2 expression.

Immunohistochemistry was performed at the Centro de Investigação Translacional em Oncologia, Instituto do Câncer do Estado de São Paulo Octavio Frias de Oliveira (ICESP), University of São Paulo, Brazil. An automated Ventana BenchMark GX system (Roche Diagnostics, Mannheim, Germany) was used in combination with an UltraView Universal DAB Detection Kit^®^ (Ventana Medical Systems, Tucson, AZ, USA), following the manufacturer’s guidelines.

Briefly, antigen retrieval was performed using a Tris-based buffer (pH 8.0; Ultra Cell Conditioning Solution, Roche Diagnostics) with heating for 30 min. The sections were then incubated with a mouse monoclonal antibody against GPx1/2 (GPx-1/2 (B-6): sc-133160; Santa Cruz Biotechnology, Dallas, TX, USA at a 1:400 dilution for 32 min. Immunoreactivity was visualized using the UltraView Universal HRP Multimer system, which contains peroxidase-conjugated secondary antibodies, in the presence of 3,3′-diaminobenzidine (DAB), resulting in a brown precipitate. The slides were counterstained with hematoxylin for 20 min.

All procedures were performed within the Ventana BenchMark GX closed system. Previously validated rat ovarian tissue samples were used as positive controls, and negative controls were obtained by omission of the primary antibody.

### 2.9. Histomorphometric and Immunohistochemical Quantification

Each H&E-stained section from each animal was subdivided into four fields, and follicles were counted at 10× magnification. Two histomorphometric parameters were simultaneously evaluated: follicular status (degenerated or mature) and the presence of corpora lutea.

For the assessment of follicular development, ovarian follicles were classified as atretic or degenerated (irregular morphology), mature (characterized by a single large antrum), or corpora lutea. Degenerated follicles were identified by the presence of a wrinkled zona pellucida and disorganized follicular cells with pyknotic nuclei, irrespective of follicular size. Viable mature follicles were defined as those without morphological signs of degeneration [33].

The GPx1/2 immunoreactivity in eight different fields per ovarian section was evaluated using a light microscope at 400× magnification.

Immunoreactivity was semi-quantitatively assessed using a scoring system ranging from 0 to 4. Staining intensity was classified as follows: 0 = no staining; 1 = weak; 2 = moderate; 3 = strong; and 4 = very strong. The proportion of stained area was also scored as follows: 0 = no staining; 1 = <25%; 2 = 25–50%; 3 = 50–75%; and 4 = >75%.

**Figure 1:**
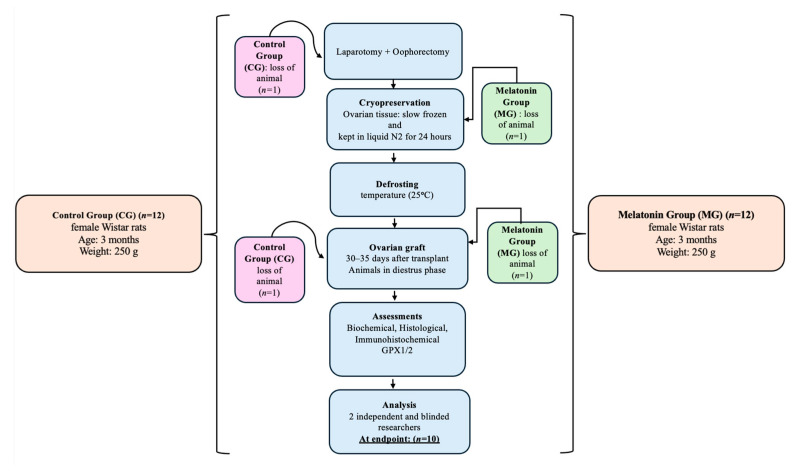
Experimental design flowchart illustrating animal allocation, ovarian tissue cryopreservation, autologous transplantation, and postoperative evaluation. A total of 24 animals were randomly allocated to the control group (CG, *n* = 12) and the melatonin-treated group (MG, *n* = 12). Due to intraoperative complications, two animals were excluded, resulting in a final sample of 20 animals (*n* = 10 per group). After transplantation, biochemical analyses were performed, including hormonal profile and VEGF-A levels as an indicator of angiogenic activity, and ovarian grafts were evaluated by histomorphological assessment (H&E staining) and immunohistochemical evaluation of GPx1/2 as an antioxidant marker. At endpoint (*n* = 10 per group) indicates the final number of animals analyzed in each experimental group after exclusions due to intraoperative complications.

In addition, quantitative morphometric analysis was performed, and the results are expressed as the percentage of GPx1/2-positive area (arbitrary units). The readings were performed by two independent investigators who were blinded to the groups (Figure 1). Photomicrographs were captured using a light microscope (Axio Imager A2, Carl Zeiss, Oberkochen, Germany) equipped with Plan-Apochromat objective lenses (10×/0.45 and 40×/0.75), coupled to a high-resolution digital camera (AxioCam MRc, Carl Zeiss, Oberkochen, Germany). Image analysis was performed using AxioVision software (version 4.6, Carl Zeiss, Oberkochen, Germany).

### 2.10. Statistical Analysis

Prior to performing statistical tests, the data distribution was assessed for normality using the Shapiro–Wilk and Smirnov–Kolmogorov tests.

Normally distributed continuous variables are expressed as the mean ± standard deviation (SD) and were compared between groups using the unpaired Student’s *t*-test or ANOVA.

Non-normally distributed continuous variables are expressed as the median and interquartile range (IQR) and were analyzed using the Mann–Whitney U test and Kruskal–Wallis test. Categorical and count data were analyzed based on their total or partial prevalence using the Chi-Square and Fisher tests.

Spearman correlation tests and linear regression analysis were performed on all continuous variables to obtain the main variables associated with melatonin supplementation during ovarian cryopreservation.

All statistical tests were two-tailed, and a *p*-value < 0.05 was considered statistically significant. No animals or samples were excluded from the analysis. Statistical analyses were performed by an investigator blinded to the group allocation using STATA 16-SE (StataCorp LLC, College Station, TX, USA).

## 3. Results

### 3.1. Estrous Cycle and Biochemical Analyses

To evaluate the functional recovery of ovarian activity after transplantation, we analyzed the dynamics of the estrous cycle and circulating hormonal parameters.

The estrous cycle was successfully characterized in all transplanted animals. Rats in the melatonin-treated group (MG) exhibited a significantly earlier return to the diestrus phase (21.45 ± 1.29 days) compared with the control group (CG; 15.52 ± 0.41 days) (unpaired Student’s *t*-test: *p* < 0.05).

All animals were euthanized during the diestrus phase to ensure hormonal standardization at the time of sample collection. As shown in Figure S1, serum FSH levels and the FSH/AMH ratio were significantly lower in the MG compared to the CG (*p* = 0.03 and *p* < 0.01, respectively). No significant differences were observed for LH, estradiol, progesterone, inhibin B, or VEGF-A levels.

Notably, the reduction in gonadotropin-related parameters (FSH level and FSH/AMH ratio), along with the trend toward lower LH levels in the melatonin-treated group, should be considered when interpreting the VEGF-A/gonadotropin ratios, as these indices reflect variations in both the numerator and denominator.

### 3.2. VEGF-A Analysis

To explore the potential effects of melatonin on angiogenic signaling, circulating VEGF-A levels and their relationship with gonadotropins were evaluated. The quantitative analysis of serum VEGF-A levels showed no statistically significant difference between the control and melatonin-treated groups; however, a non-significant increasing trend was observed in the melatonin group (98.20 ± 16.88 pg/mL vs. 80.19 ± 26.93 pg/mL; *p* = 0.1508).

**Figure 2:**
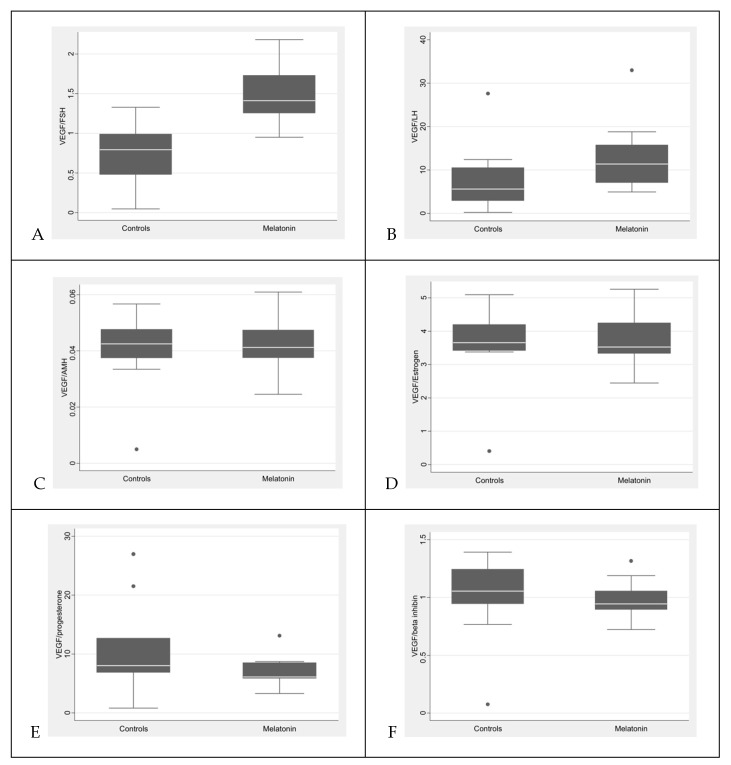
Comparison of VEGF-A/hormone ratios of the control group (CG) and melatonin-treated group (MG): (**A**) VEGF-A/FSH; (**B**) VEGF-A/LH; (**C**) VEGF-A/AMH; (**D**) VEGF-A/estradiol; (**E**) VEGF-A/progesterone; (**F**) VEGF-A/inhibin B. The MG showed significantly higher VEGF-A/FSH (*p* = 0.0007) and VEGF-A/LH (*p* = 0.0494) ratios compared to the CG. No significant differences were observed for the VEGF-A/AMH, VEGF-A/estradiol, VEGF-A/progesterone, or VEGF-A/inhibin B ratios. Data are presented as the median and interquartile range (IQR) and were analyzed using the Mann–Whitney U test. Each group included *n* = 10 animals.

In contrast, melatonin treatment resulted in a significant increase in the VEGF-A/gonadotropin ratios, specifically in the VEGF-A/FSH (*p* = 0.0007) and VEGF-A/LH (*p* = 0.0494) ratios (Table S1; Figure 2).

Overall, although absolute VEGF-A concentrations were not significantly altered, melatonin treatment was associated with significant changes in VEGF-A relative to gonadotropin levels, particularly FSH and LH.

### 3.3. Macroscopic and Histomorphometric Analyses

**Figure 3:**
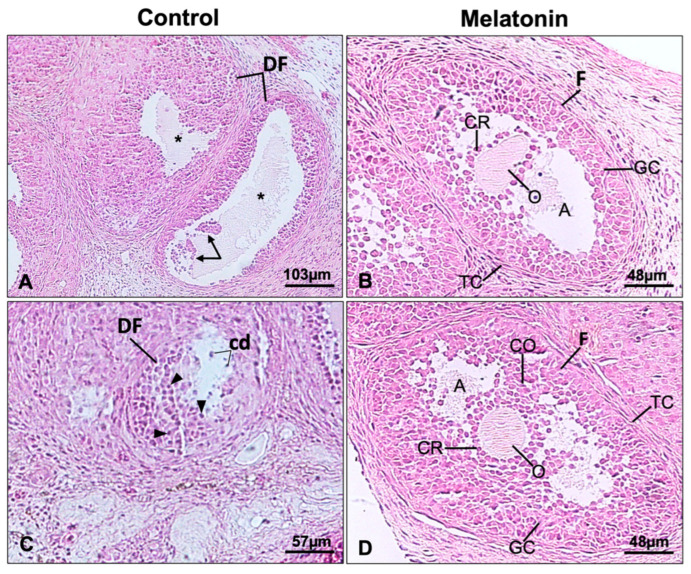
(**A**–**D**) Photomicrographs of H&E-stained rat ovarian sections. The melatonin group (**C**,**D**) shows multiple ovarian follicles at different developmental stages, whereas the control group (**A**,**B**) exhibits a few degenerating follicles (arrows). At higher magnification, a degenerating follicle is observed in the control group (**B**), with vacuolated cells (VC) in the granulosa layer and hyaline material (asterisk) indicating collapse of the zona pellucida. In contrast, the melatonin group displays a typical mature follicle with identifiable oocyte (O), granulosa cells, cumulus oophorus (CO), follicular antrum (A), corona radiata (CR), and thecal cells. Portions of corpora lutea are present in both groups.

The structural integrity of the ovarian grafts after transplantation was evaluated through macroscopic and histomorphometric analyses. Macroscopic evaluation showed that the ovarian grafts were easily identifiable and exhibited preserved morphology in both experimental groups. Histological analysis of H&E-stained sections confirmed maintenance of ovarian architecture, with the presence of follicles at different developmental stages and intact corpora lutea in both groups (Figure 3).

The quantitative histomorphometric analysis revealed differences between the experimental groups: (a) the number of degenerated/atretic follicles was significantly higher in the CG than in the MG (*p* = 0.03); (b) the number of mature follicles was higher in the MG compared with the CG but this difference did not reach statistical significance (*p* = 0.06); and (c) the number of corpora lutea and blood vessels did not differ significantly between the groups (*p* = 0.53).

### 3.4. Immunohistochemical and Morphometric Analyses of Glutathione Peroxidase

**Figure 4:**
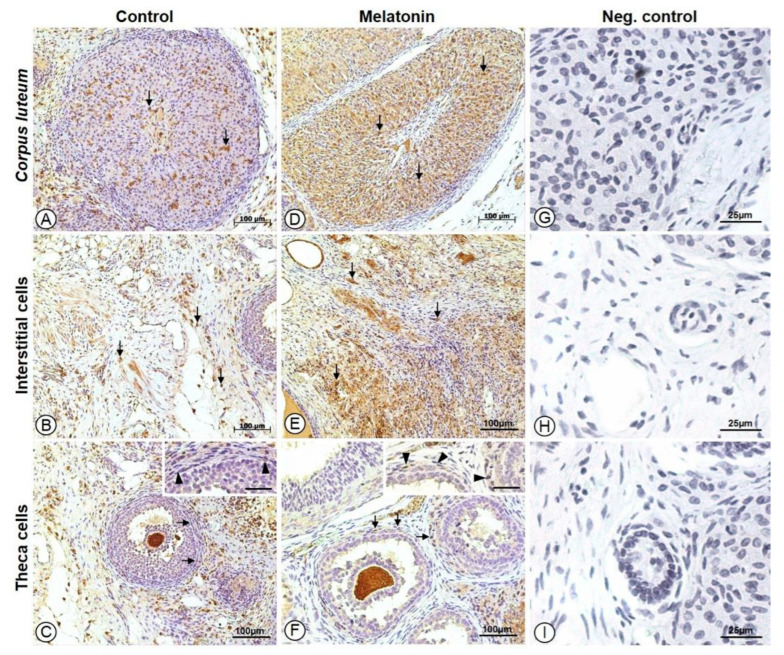
Photomicrographs of histological sections of rat ovaries subjected to immunohistochemistry for glutathione peroxidase 1/2 detection, counterstained with hematoxylin. Increased immunoreactivity for glutathione peroxidase is observed in the corpora lutea, interstitial cells, and theca cells in the melatonin-treated group (**D**–**F**) compared to the control group (**A**–**C**). Arrows and arrowheads indicate strongly immunostained cells. Insets (**C**,**F**) represent higher-magnification views of the immunostained cells indicated by arrows in the corresponding panels. Scale bars: 100 μm (**A**–**F**) and 25 μm (**G**–**I**). Negative control samples (**G**–**I**) were not incubated with the primary antibody and showed no immunostaining.

To determine whether melatonin enhances antioxidant defenses in ovarian grafts, GPx1/2 immunoreactivity was evaluated in different ovarian compartments. The immunohistochemical analysis demonstrated GPx1/2 immunoreactivity in ovarian interstitial cells, the theca interna, and corpora lutea in both groups. Qualitatively, GPx1/2 staining was more intense and widespread in the melatonin-treated group (MG) compared with the control group (CG) (Figure 4).

The melatonin-treated group showed significantly higher GPx1/2 immunoreactivity in interstitial cells, the theca interna, and corpora lutea compared with the control group (*p* = 0.012, 0.007, and 0.002, respectively) (Table 1).

Analysis of the GPx1/2-positive area revealed higher values in the melatonin-treated group in the corpora lutea and theca interna; however, these differences did not reach statistical significance (*p* = 0.126 and 0.056, respectively).

Quantitative morphometric analysis of GPx1/2 immunoreactivity showed lower interstitial cell area GPx1/2 (*p* = 0.0084) and higher luteal body intensity GPx1/2 (*p* = 0.0024) in the melatonin-treated group compared to the control group (Table 2).

### 3.5. Spearman’s Correlation Test

To investigate the relationship between antioxidant activity and angiogenic signaling, correlations between GPx1/2 expression and circulating VEGF-A levels were analyzed. Spearman’s correlation analysis revealed significant positive associations between circulating VEGF-A levels and GPx1/2 expression in the melatonin-treated group (Table S2). Specifically, higher serum VEGF-A concentrations were correlated with increased GPx1/2 immunoreactivity—increased staining intensity and/or larger positive area—in the theca interna, corpora lutea, and ovarian interstitial cells.

Overall, animals treated with melatonin that exhibited greater GPx1/2 immunoreactivity in these ovarian compartments also had higher circulating VEGF-A levels.

### 3.6. Linear Regression Analysis—VEGF-A Levels

To identify independent predictors of circulating VEGF-A levels, a multiple linear regression analysis was performed, which included hormonal and tissue antioxidant variables (Table S3). Among the hormonal parameters, AMH (*p* = 0.007), β-inhibin (*p* = 0.018), and estradiol (*p* = 0.003) were positively associated with VEGF-A concentrations, whereas LH showed a negative association (*p* = 0.027).

Regarding tissue antioxidant markers, several GPx1/2-related variables demonstrated significant associations with VEGF-A levels. The staining area and intensity of GPx1/2 in the corpus luteum, the theca interna, and interstitial cells were negatively associated with VEGF-A concentrations (*p* ≤ 0.001).

Overall, the regression model indicates that both endocrine variables and tissue GPx1/2 immunoreactivity are significantly associated with VEGF-A levels in transplanted ovarian tissue (Table S3).

### 3.7. Linear Regression Analysis—VEGF-A Levels (Melatonin-Treated Group)

To better characterize VEGF-A regulation in melatonin-treated animals, a linear regression analysis was performed on subgroups within the melatonin-treated group.

In this subgroup, circulating VEGF-A levels showed a positive association with ovarian tissue indices, including the total corpus luteum index (*p* = 0.027), theca interna index (*p* = 0.031), and total interstitial cell index (*p* = 0.019) (Table S4). The VEGF-A/FSH ratio also showed a significant association with VEGF-A levels (*p* = 0.004).

No significant negative associations with gonadotropins were observed. Overall, the model indicates that VEGF-A levels in melatonin-treated animals are associated with specific ovarian tissue indices and hormonal ratios, reflecting distinct regulation according to the hormonal and antioxidant profiles (Table S4)

## 4. Discussion

The present study’s results reinforce previous evidence for the role of melatonin as a potent antioxidant in the context of ovarian tissue cryopreservation and transplantation [8,21,34,35,36]. The significant upregulation of GPx1/2 observed in the melatonin-treated group—particularly in corpora lutea and the theca interna—suggests that melatonin effectively reduces oxidative stress in these important ovarian compartments. This finding may represent a more efficient and regulated antioxidant response, contributing to the maintenance of redox homeostasis during the post-transplant period. This interpretation is supported by the concomitant preservation of ovarian morphology and improved functional parameters observed in this group. These results are consistent with previous reports demonstrating the antioxidant capacity of melatonin in reproductive tissues [17].

Importantly, the increased GPx1/2 expression observed in this study can be interpreted from multiple complementary perspectives within the biological context of ovarian transplantation, particularly considering the stressful microenvironment the graft is exposed to after transplantation. First, ischemia–reperfusion injury and transient hypoxia are inherent characteristics of the early post-transplant period and can induce the upregulation of endogenous antioxidant enzymes, such as GPx, as an adaptive response to oxidative stress [7,8,9,10,13,14,15,16].

Second, oxidative stress plays a central role in cellular damage and reduced follicular viability during cryopreservation [7,8,10,11,12,34,35]. In this context, the protective effects of melatonin appear to be multifactorial. In addition to its well-established direct free radical-scavenging activity, melatonin has been shown to modulate the expression and activity of endogenous antioxidant enzymes, particularly the GPx family, through transcriptional and mitochondrial pathways [18,19,28,37]. Moreover, previous studies have demonstrated that melatonin can increase the activities of other key antioxidant enzymes, including superoxide dismutase (SOD) and catalase (CAT), reinforcing its broad role in activating the enzymatic antioxidant defense system [18,19,37,38,39].

Third, since both experimental groups underwent identical cryopreservation and transplantation procedures and were therefore exposed to the same oxidative conditions, the predominance of GPx1/2 upregulation in the melatonin-treated group suggests that this finding is not merely a passive response to oxidative stress. Instead, it reflects an enhanced and regulated antioxidant response mediated by melatonin.

It is important to highlight that this study also demonstrated better preservation of ovarian morphology in the group treated with melatonin, characterized by a reduced number of degenerated follicles and a greater presence of morphologically mature follicles. These histological findings corroborate the notion that melatonin not only protects against oxidative damage, but also favors post-transplant follicular development, as previously reported [17,20,21].

Theca interna cells are important for androgen production and essential for follicular steroidogenesis and estrogen synthesis [40,41,42]. These functions are regulated by luteinizing hormone (LH), which modulates the proliferation and activity of estrogen-producing granulosa cells. Excessive LH stimulation, as seen in conditions such as polycystic ovary syndrome (PCOS), can disrupt this balance and lead to altered follicular development and impaired ovulatory function [40,41,43,44]. The observed preservation of the integrity of theca interna cells in melatonin-treated grafts may therefore have broader implications for hormonal regulation and reproductive performance.

There was no significant difference between the levels of vascular endothelial growth factor-A (VEGF-A), a critical mediator of angiogenesis, in the groups in this study. Although VEGF-A is widely recognized for its central role in endothelial cell proliferation, vascular permeability, and neovascularization [23,24,45,46], the lack of a measurable effect of melatonin treatment suggests that melatonin’s protective effect in this model is primarily due to the attenuation of oxidative stress rather than angiogenic mechanisms. Future studies evaluating direct angiogenic markers and vascular density are warranted to clarify the role of melatonin in angiogenesis in this model.

In addition to its angiogenic functions, VEGF also contributes to the maintenance of vascular homeostasis and tissue repair under physiological conditions [24,45,47]. Dysregulation or overexpression of VEGF has been implicated in several pathological processes, particularly in tumorigenesis, where it promotes neoangiogenesis and increased vascular permeability [24,45,48]. Despite the lack of short-term differences in VEGF-A levels, the multifactorial actions of melatonin suggest that long-term effects on angiogenesis may arise in different physiological or pathological contexts.

This study has certain limitations, as reproductive and ovarian functional outcomes were not addressed. In addition, apoptotic pathways were not directly evaluated (e.g., using the TUNEL assay), which may limit the interpretation of the follicular degeneration observed in this model. Furthermore, no statistically significant differences in VEGF-A levels were observed, limiting conclusions about the angiogenic potential of melatonin in this context. Despite these limitations, the study provides valuable insights and lays the foundation for future clinical investigations. Its strengths include an innovative approach to the use of melatonin to minimize oxidative damage in ovarian cryopreservation and transplantation. The well-defined animal model and appropriate control and treatment groups allowed for a reliable comparison of the results. The evaluation of relevant biomarkers, such as GPx and VEGF-A, adds insights into the antioxidant and angiogenic responses induced by melatonin. The statistically significant increase in GPx expression in the melatonin group reinforces its role as a potent antioxidant, corroborating previous findings on the effects of melatonin in reproductive tissues.

## 5. Conclusions

In conclusion, this study demonstrates that melatonin supplementation enhances the antioxidant defenses of cryopreserved ovarian grafts by upregulating GPx1/2 expression, leading to reduced oxidative stress and better preservation of follicular integrity. These findings demonstrate the antioxidant potential of melatonin in the ovarian environment, as well as its possible influence on angiogenic balance (based on its effects on VEGF), with relevant implications for the preservation of reproductive function. The present study provides experimental data supporting the use of melatonin as a therapeutic strategy to mitigate the damage associated with cryopreservation in conditions that compromise ovarian health, including fertility preservation and reproductive aging.

## Figures and Tables

**Table 1:** GPx1/2 assessment of ovarian tissues from both groups using Chi-Square/Fisher Exact tests. GPx1/2 staining intensity and area were evaluated using a semi-quantitative scoring system (0–4), as described in Section 2.9. Abbreviations: CG: control group; MG: melatonin group. *n* = 10 per group.

		CG		MG		*p*-Value
		*N*	%	*N*	%	
Luteal body intensity	0	4	40.0	0	0.0	**0.002**
	1	6	60.0	1	10.0	
	2	0	0.0	3	30.0	
	3	0	0.0	5	50.0	
	4	0	0.0	1	10.0	
Internal theca intensity	0	6	60.0	0	0.0	**0.007**
	1	2	20.0	1	10.0	
	2	2	20.0	4	40.0	
	3	0	0.0	5	50.0	
	4	0	0.0	0	0.0	
Interstitial intensity	0	4	40.0	0	0.0	**0.012**
	1	4	40.0	1	10.0	
	2	2	20.0	2	20.0	
	3	0	0.0	4	40.0	
	4	0	0.0	3	30.0	
Luteal body area	0	3	30.0	0	0.0	0.126
	1	2	20.0	0	0.0	
	2	2	20.0	3	30.0	
	3	3	30.0	6	60.0	
	4	0	0.0	1	10.0	
Internal theca area	0	3	30.0	0	0.0	0.056
	1	3	30.0	0	0.0	
	2	2	20.0	3	30.0	
	3	2	20.0	6	60.0	
	4	0	0.0	1	10.0	
Interstitial cell area	0	3	30.0	0	0.0	0.165
	1	1	10.0	0	0.0	
	2	3	30.0	3	30.0	
	3	3	30.0	5	50.0	
	4	0	0.0	2	20.0	

**Table 2:** Quantitative comparison of glutathione peroxidase (GPx1/2) immunoreactivity in ovarian tissues of the control group (CG) and melatonin-treated group (MG). Data are expressed as the median and interquartile range (IQR). Statistical analysis was performed using the Mann–Whitney test; significant *p*-values are indicated in bold.

	Control Group Median (IQR)	Melatonin Group Median (IQR)	*p*-Value
Internal theca area GPx1/2	2.21 (1.13–3.08)	2.96 (2.11–3.59)	0.143
Internal theca intensity GPx1/2	1.68 (1.09–2.38)	2.36 (2.06–2.89)	0.1573
Interstitial cell area GPx1/2	3.39 (3.08–3.62)	2.47 (2.31–3.02)	**0.0084**
Interstitial cell intensity GPx1/2	1.12 (1.03–2.32)	3.10 (2.45–3.47)	0.0652
Luteal body area GPx1/2	2.55 (1.13–3.48)	2.96 (2.11–3.47)	0.0654
Luteal body intensity GPx1/2	1.13 (1.03–1.27)	2.69 (2.06–3.17)	**0.0024**

## Data Availability

All original contributions presented in this study are included in the article/Supplementary Materials. Further inquiries can be directed to the corresponding author.

## Supplementary Materials

The following supporting information can be downloaded at: https://www.mdpi.com/article/10.3390/antiox15050551/s1. Figure S1: The biochemical data in the animals that underwent transplantation, compared melatonin group (MG) with control group (CG). The FSH levels in the MG were lower than those in the CG *p* = 0.03. The FSH/AMH ratios were multiplied by 100, resulting in the following values: GM which was significantly lower than that of CG *p* < 0.01. Differences in LH, estradiol, progesterone, and inhibin B were not found between the groups. VEGF-A levels did not show a statistically significant difference between the groups. Abbreviations: A: FSH (follicle-stimulating hormone); B: LH (luteinizing hormone); C: AMH (anti-Mullerian hormone); D: inhibin B; E: estradiol; F: progesterone; G: VEGF; H: FSH/AMH *100 ratio; Table S1: Comparison of quantitative data between VEGF and biochemical analyses. Student *t*-test; Table S2: Spearman’s correlation test in the Melatonin group; Table S3: Linear regression—VEGF-A; Table S4: Linear regression—VEGF—Melatonin group.

## Abbreviations

The following abbreviations are used in this manuscript:

AMH	Anti-Müllerian hormone
CAT	Catalase
CG	Control group
CL	Corpus luteum
DAB	3,3′-Diaminobenzidine
DMSO	Dimethyl sulfoxide
ELISA	Enzyme-linked immunosorbent assay
FSH	Follicle-stimulating hormone
GPx	Glutathione peroxidase
GPx1/2	Glutathione peroxidase isoforms 1 and 2
H&E	Hematoxylin and eosin
LH	Luteinizing hormone
MG	Melatonin group
ROS	Reactive oxygen species
SOD	Superoxide dismutase
SOD2	Superoxide dismutase 2
VEGF	Vascular endothelial growth factor
VEGF-A	Vascular endothelial growth factor A
ZT	Zeitgeber time
