# Supplementary material for: Melatonin Enhances Glutathione Peroxidase Activity and Improves Antioxidant Defense in Cryopreserved Ovarian Transplants: A Rat Model Study
Source: Antioxidants (Basel). 2026 Apr 26;15(5):551. doi: 10.3390/antiox15050551 (PMC13203636; doi:10.3390/antiox15050551)
Supplement: Supplementary file 1 [file antioxidants-15-00551-s001.zip › antioxidants-4198333-supplementary.pdf]

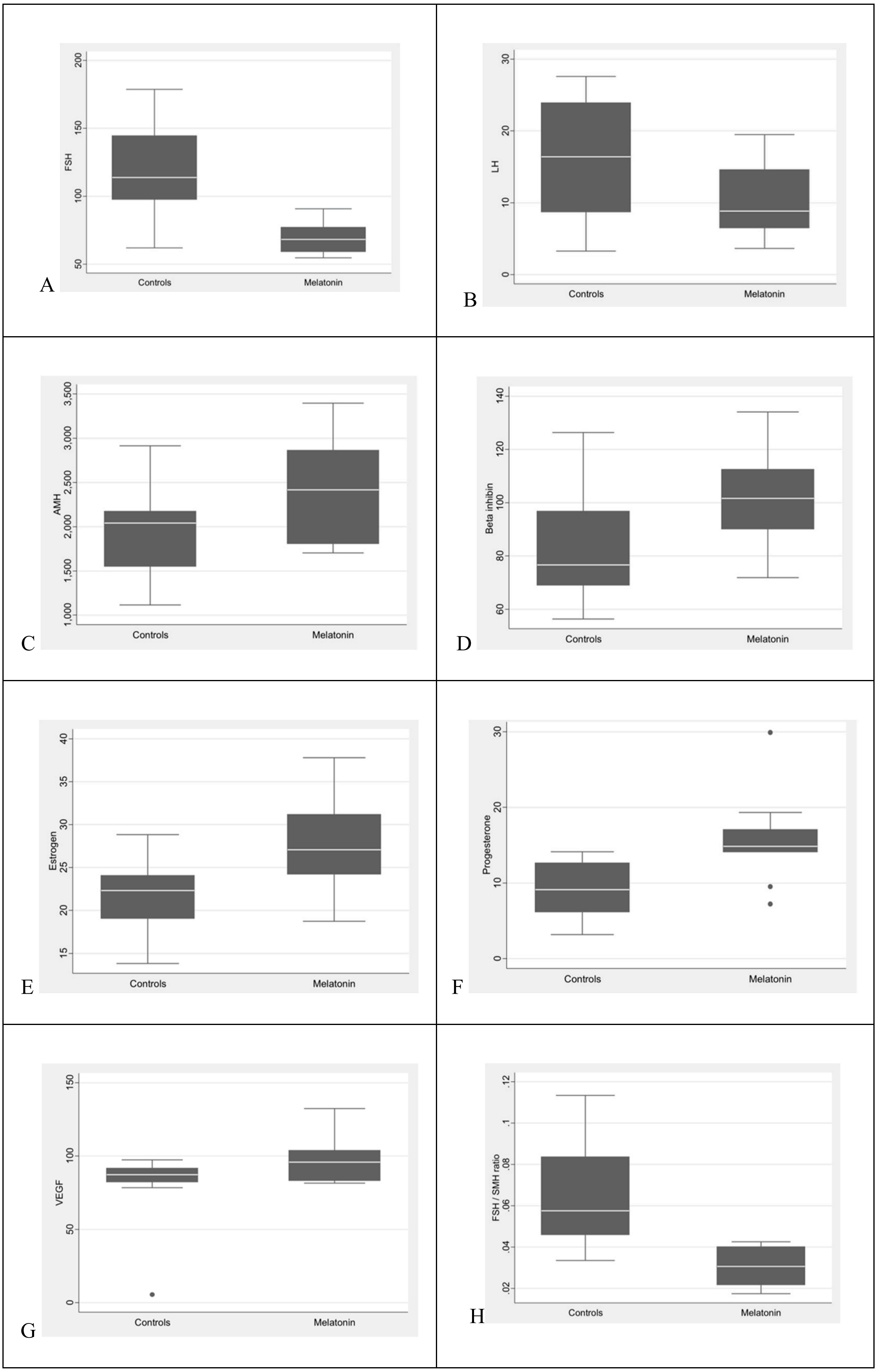

Figure S1. The biochemical data in the animals that underwent transplantation, compared melatonin group (MG) with control group (CG). The FSH levels in the MG were lower than those in the CG p = 0.03. The FSH/AMH ratios were multiplied by 100, resulting in the following values: GM which was

significantly lower than that of CG $p < 0.01$. Differences in LH, estradiol, progesterone, and inhibin B were not found between the groups. VEGF-A levels did not show a statistically significant difference between the groups. Abbreviations: **A:** FSH (follicle-stimulating hormone); **B:** LH (luteinizing hormone); **C:** AMH (anti-Mullerian hormone); **D:** inhibin B; **E:** estradiol; **F:** progesterone; **G:** VEGF; **H:** FSH/AMH *100 ratio

Table S1: Comparison of quantitative data between VEGF and biochemical analyses. Student T-test

	**Control**					**Melatonin**					**P**
	**Med**	**Sd**	**Median**	**Q1**	**Q3**	**Med**	**Sd**	**Median**	**Q1**	**Q3**	
**VEGF**	80.19	26.93	87.27	82.35	91.61	98.20	16.88	95.80	83.35	103.80	0.1508
**VEGF/AMH**	0.04	0.01	0.04	0.04	0.05	0.04	0.01	0.04	0.04	0.05	0.9397
**VEGF/beta inhibin**	1.01	0.38	1.05	0.95	1.24	0.99	0.17	0.94	0.90	1.05	0.2899
**VEGF/Estrogen**	3.61	1.28	3.65	3.42	4.20	3.69	0.76	3.53	3.34	4.25	0.5453
**VEGF/FSH**	0.73	0.36	0.79	0.48	0.99	1.47	0.37	1.41	1.26	1.73	0.0007*
**VEGF/LH**	7.94	7.82	5.60	2.99	10.52	13.04	8.38	11.39	7.10	15.72	0.0494*
**VEGF/progesterone**	10.94	7.80	8.03	6.92	12.67	6.98	2.62	6.11	5.88	8.52	0.1736
**FSH / AMH**	0.07	0.03	0.06	0.05	0.08	0.03	0.01	0.03	0.02	0.04	0.0007

Abbreviations: CG: control group; MG: melatonin group; VEGF: Vascular Endothelial Growth Factor; GPx: Glutathione Peroxidase; FSH: follicle-stimulating hormone; AMH: anti-müllerian hormone; LH: luteinizing hormone. (*) significant p values.

Table S2- Spearman's correlation test in the Melatonin group

Internal Theca Index	Total interstitial cell index	0.9179
Internal theca intensity GPx	Luteal body intensity GPx	1
total interstitial cell index	Total intestitial cell index/VEGF	0.9103
Internal theca index/VEGF	Internal theca intensity GPx	0.7651
Internal theca index/VEGF	interstitial cell intensity GPx	0.8056
Internal theca index/VEGF	Luteal body area GPx	0.7981
Internal theca index/VEGF	total interstitial cell index	0.7597
Luteal body area GPx	Luteal body intensity GPx	0.8291
Luteal body area GPx	Total intestitial cell index/VEGF	0.8718
AMH	Estrogen	0.8789
AMH	LH	-0.8274
AMH	VEGF/AMH	-0.7738
AMH	VEGF/LH	0.7656
Estrogen	VEGF/LH	0.8718
FSH	VEGF/FSH	-0.7274
LH	VEGF/AMH	0.7636
LH	VEGF/LH	-0.8078
Progesterone	VEGF/progesterone	-0.7941
VEGF	VEGF/FSH	0.8214
VEGF/AMH	VEGF/Estrogen	0.8751
VEGF/beta inhibin	VEGF/progesterone	0.7273

Spearman correlation analysis in the melatonin-treated group, showing significant associations between biochemical parameters and the expression of VEGF-A and GPx1/2 in rat ovarian tissue, presented in decreasing order of

statistical significance. Abbreviations: VEGF: Vascular Endothelial Growth Factor; GPx: Glutathione Peroxidase; FSH: follicle-stimulating hormone; AMH: anti-müllerian hormone; LH: luteinizing hormone.

Table S3 - Linear regression - VEGF -A

	Coef	95%CI min	95%CI max	p
**AMH**	0.025	0.008	0.041	0.007
**Beta inhibin**	0.574	0.112	1.035	0.018
**Progesterone**	1.669	-0.086	3.425	0.061
**Estrogen**	2.603	0.991	4.215	0.003
**FSH**	-0.185	-0.486	0.116	0.213
**LH**	-1.549	-2.901	-0.198	0.027
**Internal theca area GPx**	-5.641	-7.630	-3.652	0.001
**Internal theca Index**	3.610	1.113	6.107	0.008
**Internal theca intensity GPx**	1.185	-11.841	14.211	0.846
**interstitial cell area GPx**	-2.658	-3.602	-1.715	0.001
**interstitial cell intensity GPx**	-2.625	-3.566	-1.684	0.001
**Luteal body area GPx**	-5.143	-7.399	-2.888	0.001
**Total intestitical cell index/VEGF**	-1.262	-1.797	-0.727	0.001
**VEGF/AMH**	1258.258	513.359	2003.158	0.002
**VEGF/beta inhibin**	48.657	15.455	81.859	0.006
**VEGF/Estrogen**	16.067	7.835	24.299	0.001
**VEGF/FSH**	34.032	18.720	49.345	0.001
**VEGF/LH**	1.443	0.223	2.663	0.023

Abbreviations: CI: confidence interval; FSH: follicle-stimulating hormone; AMH: Anti-Mullerian hormone; LH: luteinizing hormone; VEGF: Vascular Endothelial Growth Factor; GPx: Glutathione Peroxidase.

Table S4. Linear regression - VEGF - Melatonin group.

	Coef	95%CI min	95%CI máx.	P
**VEGF/LH**	0.961	-0.481	2.403	0.163
**VEGF/FSH**	37.801	16.403	59.199	**0.004**
**VEGF/AMH**	518.182	-803.033	1839.398	0.392
**VEGF/ Estrogen**	6.222	-11.051	23.496	0.43
**VEGF/Progestagen**	0.436	-4.795	5.666	0.852
**VEGF/ Beta inhibin**	29.136	-49.060	107.332	0.415
**Luteal body area**	1.598	-16.497	19.692	0.844
**Luteal body intens**	0.072	-20.768	20.912	0.994
**Internal theca intens**	0.072	-20.768	20.912	0.994
**Internal theca area**	-10.138	-28.511	8.236	0.239
**Interstic cell intens**	-2.633	-16.146	10.879	0.665
**Interstic cell area**	3.371	-16.543	23.284	0.706
**Total body luteal index**	2.927	0.424	5.430	**0.027**
**Total body luteal index VEGF**	180.106	-208.114	568.326	0.316
**Internal theca index**	3.997	0.456	7.538	**0.031**
**Internal theca index VEGF**	179.952	-385.063	744.967	0.484
**Total interstic cell index**	2.834	0.593	5.075	**0.019**
**Total interstic cell index VEGF**	2.059	-1.686	5.804	0.24

Abbreviations: CI: confidence interval; FSH: follicle-stimulating hormone; AMH: Anti-Mullerian hormone; LH: luteinizing hormone; VEGF: Vascular Endothelial Growth Factor;.
